# Medical and Physical Intelligence

**Published:** 1826-02

**Authors:** 


### MEDICAL AND PHYSICAL INTELLIGENCE.

### PATHOLOGY.

1. *Softening of the Stomach.*—AMONG the publications sent to us during the present month, is one entitled "De Gastromalacia et Gastropathia Infantum; auctore F. X. RAMISCH. Prague, 1824."—Considerable attention has lately been excited by the frequent detection of a softened state of the stomach, or complete laceration of that organ, in children. The German practitioners, particularly, have paid great attention to the subject, and much interesting information and research will be found in Dr. GAIRDNER's Paper, in the Transactions of the Medico-Chirurgical Society of Edinburgh. In our own Journal, also, for December 1824, and in the Repository for August 1815, Mr. NORTH has published some observations upon the same subject. The latter case is frequently referred to by Dr. Ramisch.

When *Gastromalacia*, or the softened state of the membranes of the stomach, is detected upon examination, the infant has generally exhibited, during life, anomalous symptoms of disturbance of the intestinal canal. Vomiting or diarrhœa, or both, are common, together with lingering febrile action, and much prostration of strength. To the symptoms supposed to be indicative of the mischief going on in the stomach, the term Gastropathia is applied by the author of the little book we are noticing. A great variety of names have been used by different foreign authorities to designate this destruction of the stomach, with which the continental practitioners are so much more conversant than ourselves. Mr. HUNTER considered it to be "digestion of the stomach after death." (*Philos. Transactions*, 1772, vol. lxii. p. 447.) Several pages of reference to the works and papers of different writers are given, and will be consulted with much advantage by those who are particularly interested upon the subject.

The author has bestowed considerable pains to establish a diagnosis between the symptoms which precede the organic destruction of the stomach, and other infantile derangements of less serious import. In our opinion, he has failed in his laudable attempt, which he appears to have prosecuted with singular industry, if we may judge from the immense number of works he has quoted. The symptoms which are supposed particularly to indicate this affection, are such as are commonly exhibited in most cases of gastric disturbance. In their progress, they are not unusually complicated with others arising from disease of other parts.

This total or partial destruction of a portion of the stomach in infants has sometimes been detected after death, when the patient had not shown any symptoms during life which could have excited any suspicion of the nature of the malady.

Upon dissection, we generally find the cardiac extremity of the stomach dissolved, as if by some caustic preparation: in a few instances, the pyloric has been the suffering part. The size of the aperture in the stomach, and the colour of the edges of the opening and of the surrounding parts, are very various.

*Prognosis.*—Our opinion as to the termination of this disease must be at all times doubtful, although some cases have certainly terminated in recovery, when the assistance of the practitioner has been required at the commencement of the gastric disturbance.

The treatment must necessarily vary with the particular disposition of the infant. In some cases we shall have to contend with inflammatory action; in others, with pure debility of the system. Hence the conflicting opinions, which our author has collected with more labour than practical profit, upon the therapeutical part of the subject.

---

### THERAPEUTICS.

2. *Effects of Strychnia in Epilepsy.*—A young man, aged thirty-two, had been subject to epilepsy from the age of twelve, having ten or twelve fits in the day. Every means of cure had been attempted, excepting the administration of strychnia. The sixth part of a grain was given by Dr. Brofferio, night and morning. For three days, the only observable effect of the medicine was, that the fits were shorter and less violent. A quarter-of-a-grain dose was then prescribed. The patient slept all night, and had no fit for the thirteen following days; but, on waking in the mornings, he complained of giddiness, and was not able to move the lower extremities: however, after dinner, all these symptoms disappeared. After these thirteen days of amendment, the patient experienced in the morning four fits, which were of short duration. The strychnia was now given in the dose of half a grain, and the fits ceased for twelve days; at the end of which time they were renewed, very slightly indeed, but enough to induce the Doctor to administer the medicine in doses of one grain. There was now an intermission of five days; but at the end of that time a very violent fit came on, in which the patient died. (*Repertorio Med. Chir. de Torino,* Luglio.)

3. *Curious Case of Dyspnœa, cured by the Application of a Bandage.*—" The relatives considered the case as quite hopeless: indeed, the mother said, as we were going towards the bed-room, ' You are quite too late, Sir; you can do no good.' Upon entering the room, I found the respiration so very laborious, and at times so interrupted, that I was of the same opinion. Bleeding and various other remedies had been tried during the three days preceding the time of my visit, without any good effect whatever. I directed the mother to make a long and broad bandage, which we applied pretty tight round the thorax and a good part of the abdomen. The respiration gradually became more easy; and, in the course of twenty-four hours, it became so easy, that the poor patient could walk gently about the house.

"I leave your readers to form their own conjectures respecting the cause and cure of the disease. I may add, that the young woman, who was about twenty years of age, had enjoyed good health until about a week before I saw her, when she complained at times of more or less difficulty of breathing." (*Private Letter from* Dr. GILBY.)

*Clifton; October 31st,* 1825.

4. *Marvellous Effects of Acupuncturation.*—This remedy, after having made a little impression in this country, has been enthusiastically received in France, and at length passed into Italy, where it has obtained an advocate in the person of Dr. CARBARO, who has published a long Essay upon this subject, which contains some startling propositions. He says that these needles may be passed with impunity through the largest blood-vessels or nerves. He advises them not to be kept in above fifteen minutes at furthest; and, if the pain suddenly shifts from the part where the punctures are made to some other, we are directed to follow and attack it in its new situation. The cases which our author relates of cures performed by acupuncturation, include acute rheumatism, pleurisy, and convulsions. In pleurisy, we are told that three needles were employed, which were fairly pushed into the cavity of the chest. All these cures took place in a few minutes.

Dr. Carbaro next relates the result of some experiments made upon kittens, in the presence of several people, whose names are mentioned, and who are called upon to testify to their accuracy. Three kittens were plunged into cold water, and kept there until all signs of life was extinct; which being made evident to the bystanders, the Doctor next proceeded to endeavour to resuscitate them by frictions and other means; but in vain. At length, recourse was had to the acupuncture needles, which he passed through the heart, having previously cut off the hair from the chest opposite to the situation of this organ. At the end of fifteen minutes, the needle situated in the heart was observed to move. This leaping or moving went on increasing rapidly, and was followed by motions of the anterior extremities; then by respiration, by crying, and finally by the movement of the whole body.

Two other experiments are related, but they are too long for insertion; and, finally, the paper concludes with the successful application of the needles in a case of erysipelas of the head, with a threatening of phrenitis. "*It was beautiful to see,*" says the Doctor, "*the red colour disappearing a minute and a half after the operation; so that, in twelve minutes, the face had acquired its natural colour, the volume of the head had decreased, the eyes opened, the delirium vanished, together with the fever, and the cure was effected with the rapidity of lightning!*" (*Annali Universali,* Agosto.)

### STATISTICAL MEDICINE.

5. *Table of Admissions and Deaths at the Small-Pox Hospital during fifty Years, viz. from* 1776 *to* 1825, *inclusive: with the Rate of Mortality per Cent. in each year, (omitting Fractions.)* (Dr. GREGORY's *Report.*)

| *Year.* | *Admissions.* | *Deaths.* | *Rate of Mortality per Cent.* | *Year.* | *Admissions.* | *Deaths.* | *Rate of Mortality per Cent.* |
|---|---|---|---|---|---|---|---|
| 1776 | 374 | 80 | 21 in 100 | 1801 | 177 | 55 | 31 in 100 |
| 1777 | 497 | 125 | 25 .... | 1802 | 175 | 68 | 39 .... |
| 1778 | 269 | 74 | 27 .... | 1803 | 98 | 22 | 22 .... |
| 1779 | 351 | 136 | 38 .... | 1804 | 48 | 8 | 17 .... |
| 1780 | 175 | 53 | 30 .... | 1805 | 280 | 97 | 35 .... |
| 1781 | 646 | 257 | 40 .... | 1806 | 100 | 35 | 35 .... |
| 1782 | 122 | 52 | 42 .... | 1807 | 172 | 48 | 28 .... |
| 1783 | 382 | 121 | 32 .... | 1808 | 128 | 36 | 28 .... |
| 1784 | 351 | 127 | 36 .... | 1809 | 146 | 41 | 28 .... |
| 1735 | 314 | 108 | 34 .... | 1810 | 149 | 51 | 34 .... |
| 1786 | 175 | 57 | 29 .... | 1811 | 94 | 22 | 23 .... |
| 1787 | 362 | 106 | 29 .... | 1812 | 144 | 54 | 37 .... |
| 1788 | 142 | 49 | 34 .... | 1813 | 69 | 18 | 26 .... |
| 1789 | 285 | 107 | 38 .... | 1814 | 79 | 26 | 39 .... |
| 1790 | 192 | 68 | 35 .... | 1815 | 101 | 34 | 34 .... |
| 1791 | 277 | 89 | 32 .... | 1816 | 141 | 29 | 20 .... |
| 1792 | 188 | 58 | 30 .... | 1817 | 160 | 48 | 30 .... |
| 1793 | 358 | 134 | 37 .... | 1818 | 58 | 14 | 24 .... |
| 1794 | 213 | 72 | 33 .... | 1819 | 193 | 61 | 32 .... |
| 1795 | 180 | 48 | 27 .... | 1820 | 142 | 34 | 25 .... |
| 1796 | 447 | 148 | 33 .... | 1821 | 117 | 49 | 42 .... |
| 1797 | 51 | 14 | 27 .... | 1822 | 194 | 57 | 29 .... |
| 1798 | 265 | 89 | 33 .... | 1823 | 151 | 37 | 24 .... |
| 1799 | 151 | 43 | 28 .... | 1824 | 199 | 54 | 27 .... |
| 1800 | 250 | 68 | 27 .... | 1825 | 419 | 120 | 29 .... |
| 25 Years | 7017 | 2277 | 32½ .... | 25 Years | 3743 | 1118 | 30 .... |

6. ***Table of the Numbers Vaccinated at the Small-Pox Hospital during twenty Years, (divided into four Periods, of five Years each.)***

| *Period.* | *Years.* | *Numbers Vaccinated annually.* | *Numbers Vaccinated in five Years.* |
|---|---|---|---|
| First Period | 1806 | 946 | 7004 |
| | 1807 | 1577 | |
| | 1808 | 1246 | |
| | 1809 | 1515 | |
| | 1810 | 1720 | |
| Second Period | 1811 | 1458 | 9339 |
| | 1812 | 1939 | |
| | 1813 | 1831 | |
| | 1814 | 1671 | |
| | 1815 | 2440 | |
| Third Period | 1816 | 2313 | 13,348 |
| | 1817 | 3124 | |
| | 1818 | 2161 | |
| | 1819 | 3328 | |
| | 1820 | 2422 | |
| Fourth Period | 1821 | 2842 | 16,666 |
| | 1822 | 3368 | |
| | 1823 | 3129 | |
| | 1824 | 3324 | |
| | 1825 | 4003 | |
| Total vaccinated in 20 years, at the Small-Pox Hospital | | | 46,357 |

*(Ibid.)*

7. ***Table of the Diseases, Casualties, &c. within the City of London and Bills of Mortality, from December* 14, 1824, *to December* 13, 1825; *according to the Reports made to the King and to the Lord Mayor, by the Company of Parish Clerks.***

DISEASES.

| | | | | | |
|---|---|---|---|---|---|
| Abscess | 89 | Fever (Typhus) | 86 | Palpitation of the Heart | 2 |
| Age and Debility | 1528 | Fever Intermittent, or Ague | 1 | Palsy | 116 |
| Apoplexy | 317 | Fistula | 5 | Paralytic | 35 |
| Asthma | 816 | Flux | 10 | Pleurisy | 8 |
| Bedridden | 2 | Gout | 26 | Rheumatism | 19 |
| Bile | 6 | Hæmorrhage | 31 | Scrofula | 10 |
| Cancer | 95 | Hernia | 20 | Small-pox | 1299 |
| Consumption | 5062 | Hooping-Cough | 420 | Sore Throat or Quinsey | 15 |
| Convulsions | 2662 | Hydrophobia | 4 | Spasm | 58 |
| Croup | 82 | Inflammation | 2196 | Still-born | 904 |
| Diarrhœa | 8 | Inflammation of the Liver | 130 | Stone | 20 |
| Dropsy | 313 | Insanity | 198 | Stoppage in the Stomach | 21 |
| Dropsy in the Brain | 751 | Jaundice | 27 | Suddenly | 125 |
| Dropsy in the Chest | 65 | Jaw-locked | 2 | Teething | 408 |
| Dysentery | 5 | Lethargy | 1 | Thrush | 59 |
| Enlargement of the Heart | 12 | Livergrown | 3 | Tumor | 7 |
| Epilepsy | 40 | Measles | 743 | Venereal | 5 |
| Eruptive Diseases | 10 | Miscarriage | 1 | | |
| Erysipelas | 20 | Mortification | 279 | Total of Diseases | 20,672 |
| Fever | 809 | | | | |

CASUALTIES.

| | | | | | |
|---|---|---|---|---|---|
| Broken Heart...... | 2 | Frighted .......... | 2 | Shot .............. | 1 |
| Broken Limbs ..... | 1 | Killed by Falls and several other Accidents .......... | 95 | Stabbed .......... | 1 |
| Burnt ............ | 36 | Killed by Fighting.. | 1 | Strangled ........ | 1 |
| Choked ........... | 1 | Murdered ........ | 1 | Suffocated ........ | 3 |
| Drowned.......... | 139 | Poisoned.......... | 5 | Suicides .......... | 42 |
| Excessive Drinking . | 3 | Scalded .......... | 5 | | |
| Executed* ....... | 4 | | | Total of Casualties . | 354 |
| Found Dead ...... | 11 | | | | |

CHRISTENED.

| | | |
|---|---|---|
| Males ....12,915 | In all, 25,634 | |
| Females ..12,719 | | |

BURIED.

| | | |
|---|---|---|
| Males ....10,825 | In all, 21,026 | |
| Females ..10,201 | | |

Whereof have died,

| | | | |
|---|---|---|---|
| Under Two Years of Age | 6419 | Fifty and Sixty .......... | 1746 |
| Between Two and Five .. | 2061 | Sixty and Seventy ...... | 1740 |
| Five and Ten............ | 867 | Seventy and Eighty ...... | 1568 |
| Ten and Twenty ........ | 877 | Eighty and Ninety ...... | 622 |
| Twenty and Thirty ...... | 1485 | Ninety and a Hundred .. | 72 |
| Thirty and Forty ........ | 1698 | A Hundred ............ | 1 |
| Forty and Fifty.......... | 1831 | A Hundred and One .... | 1 |

Increased in the Burials this Year, 781.

### SURGERY.

8. *Re-union of a Nose, which had been completely separated.*—Notwithstanding the many experiments that have been instituted, and the various facts that have been recorded, in proof of the power which nature possesses of re-uniting parts which have been completely and for some time separated from the body, there is still a disposition, on the part of some practitioners, to smile with incredulity at these statements, and to omit to take advantage of the opportunities which accident may afford them of putting their accuracy to the test. Within the circle of our own knowledge, for instance, a case lately occurred, in which the patient was suffered to remain permanently maimed, from this unwarrantable scepticism. A man had two fingers completely separated from his hand, near the metacarpal extremities, by a sharp and clean axe. A surgeon (by title) saw the patient in less than five minutes. The stumps were dressed *secundum artem*, but no attempt was made to re-unite the yet warm fingers to the separated part. Success would, in all probability, have attended the experiment; and the man would have been enabled to support his family, instead of becoming a charge upon his parish,—which is now, in fact, the case.

The following abstract of an instance in point we take from one of the best German Journals of the day:—

An unfortunate tailor, by the name of Gruzlewski, seated himself in a window, one wing of which he had opened. A sudden and violent gust of wind shut it with considerable force, and a part of the glass which was broken carried off a great portion of the man's nose. The separated piece was about the length of a finger, and the whole breadth of the nose. It fell from the second story of the house into the street.

* There have been executed within the Bills of Mortality, 15; only 4 have been reported as such.

The circumstance occurred about seven o'clock in the evening. A surgeon was immediately sent for, and he was satisfied with merely applying a plaster. Another surgeon, however, was consulted two hours after the accident. He sought for the nose with a candle in the street, and placed it in its natural situation. In a few days it had united, and regained its warmth and sensibility. The only mark of the accident which remains perceptible is a small, narrow, red scar.

It is observed, that the magistrates would testify the truth of this relation, if it were considered necessary.

A similar case is also recorded in the same Journal, in which complete union took place, where the nose had been entirely separated. (*Journal der Chirurgie und Augen-Heilkunde*, von GRAFE und WALTHER; band 7, heft 4.)

For much interesting information upon the subject of the re-union of divided parts, we refer our readers to a publication of WIESMANN, " De Coalitu partium a reliquo Corpore prorsus disjunctarum."

9. *Gangrena Senilis.*—DUPUYTREN recommends the application of leeches in this form of complaint. By their frequent application, he cured an old woman, of sixty years of age, in the Hôtel Dieu. The usual sedative, antispasmodic, tonic, and antiseptic means, had been tried in vain. The authority of this eminent surgeon is doubtless to be received with much attention; but yet we may be allowed to doubt, not from any abstract opinions upon the subject, but from attentive observation, whether there are many cases of true gangrena senilis in which we can venture upon debilitating means of any kind.

---

### MIDWIFERY.

10. *Case of Abdominal Pregnancy.*—CHARLOTTE N—, thirty-six years of age, of a strong constitution, mother of six children, suffered, for some years, from all the presumptive symptoms of pregnancy; added to which, there was a constant pain at the lower and left side of the belly. This pain was at times very severe. Menstruation was not suppressed, but a discharge of blood from the vagina generally occurred once in six or eight days. Nevertheless, the patient's health was not much disturbed. About six weeks prior to the presumed period of her pregnancy, Mrs. N— had been washing, and of course had bent her body forwards. This motion was followed by a sound as if something had broken in the abdomen; and at the same time a tumor, the size of two fists, became apparent below the umbilicus on the right side. The patient fell down senseless, and was carried home. She felt for six weeks dull pains: after this period, labour-pains came on, and a midwife was sent for. For two nights these pains continued, and the uterus was sufficiently dilated to admit a finger. The tumor before spoken of had disappeared on the commencement of the pains; and, on the third day, these ceased altogether; the patient only complaining of some weakness, and a distention of the abdomen.

Things remained in this state for two or three months; the menses were regular, but not abundant. At length, symptoms of a fresh pregnancy occurred, which went on regularly to the seventh month; at which time the parietes of the abdomen assumed a bluish tint, and the slightest movement discovered a fluctuation in the cavity. At length, at the usual period, the patient was delivered of a large male infant, which she nourished for fifteen days, but which died at the end of nine weeks. The mother lost her milk.

The moment she ceased to give suck, she lost all her strength; complained of a fixed pain in the belly, with fever, and agitation during the night; and her emaciation was extreme. A tumor, similar to that which had appeared before, showed itself an inch below the navel: it was the size of a hen's egg, and it broke by two small orifices, discharging some pus. A surgeon was sent for, who enlarged the opening, and a considerable quantity of purulent sanies escaped, with portions of skin and hair; and, on feeling the tumor, hard substances like bone were felt. Another eminent surgeon was consulted, who found the patient sinking under hectic fever. The uterine orifice was firmly closed, and nothing demonstrating the presence of a fœtus was traceable there: but it was evident that one existed in the abdomen, and it was determined to extract it by an operation, which was thus performed:—

The bladder and rectum being both emptied, the patient was placed on a table, each foot resting upon a chair. An incision, of fifteen lines in length, was then made in the linea alba, two and a half inches above the umbilicus, and the peritoneum divided with the greatest precaution; then, passing the index and middle finger within the opening from above downwards, the operator raised the abdominal parietes, and then, passing the bistoury in the groove between the two fingers, the incision was continued through the linea alba to nine lines above the pubes. The exit of the intestines was prevented by means of a linen cloth, oiled, which an assistant stretched over the wound as soon as the parts were divided. This being done, a fœtus at the full period was perceived, situated to the right of the uterus; the head corresponding to the same groin, the back to the iliac fossa, and the feet, slightly bent, towards the right hypochondrium; the left shoulder at the inferior part of the umbilical region, precisely where the fistulous opening above mentioned had been. The dead fœtus was removed; the chord was found wrapped round the fundus of the uterus, then turned upon its left side, and was buried in a vascular network in a state of suppuration, probably the remains of the placenta, which was found between the epiploon. This pus was absorbed by means of a sponge; the wound was brought together by sutures, and dressed.

Inflammatory symptoms threatened the life of the patient for some days: it was only at the end of a week that hopes of her recovery were entertained, which were at length realised. She quitted her bed on the fifty-fifth day. The operator's name was M. BUTH. HUFELAND'S *Journal*, May.)

---

### CHEMISTRY.

11. *Analysis of Opium.*—M. ROBIQUET presented to the Royal Academy of Medicine some observations on the new analysis of opium, by the action of watery solutions of muriate of soda, according to the method of M. ROBINET. This chemist does not think that the organic alkalies exist in vegetables combined with ordinary acids, but rather with particular substances which fulfil the same functions; such as colouring principles. The acids of vegetables would combine, by preference, with earths, or the fixed alkalies of plants.

Having repeated the experiments of M. Robinet to separate the morphia of opium, he obtained a pitchy precipitate; and the supernatent liquor, when filtered, being boiled and treated with ammonia, precipitated very little morphia: the remainder of the liquor let fall a grained precipitate, which, when purified and examined, was found to be a *muriate of morphia.* M. Robiquet is then of opinion that the pretended *codeate* of morphia is only a muriate, easily recognised by the vapours which it exhales with concentrated sulphuric acid, as well as by the precipitate which it yields with nitrate of silver. The muriate of soda, announced by M. Robinet, results from a change of bases, very common in complex combinations. M. PELLETIER observes, that he has also made out that the codeate is merely a muriate. (*Archives Generales,* November.)

---

### MISCELLANEOUS.

12. *Prize Essay.*—The Royal Medical Society of Edinburgh propose, as the subject of their Prize Essay, the following questions:—

1st, What is the respective agency of the veins and the lymphatics, in the process of absorption?

2d, By what means, or mechanism, do these vessels accomplish this process? What are the proofs which show that the substances absorbed are taken up by open mouths or orifices, or pass through the coats in the manner of imbibition or transudation?

3d, Is there any reason to believe that the individual animal tissues possess a distinct power of absorption, or that this process is influenced by the nature of the animal tissues?

The sum of twenty guineas, or a medal, or a set of books of that value, will be given to the author of the best Dissertation on the subject proposed by the Society; for which all men of science are invited to compete.

The Dissertations may be written in English, French, or Latin; and must be transmitted to the Secretary on or before the 1st of December, 1826; and the adjudication of the prize will take place in the last week of the month of February following.

To each Dissertation must be prefixed a motto; which must likewise be written on the outside of a sealed packet, containing the name and address of the author. No Dissertation will be received with the author's name affixed; and all Dissertations, except the successful one, will be returned (if desired,) with the sealed packet unopened.

### METEOROLOGICAL JOURNAL,

*From December 20, 1825, to January 19, 1826.*

| | MOON. | Rain gauge | THERM. | | | BAROM. | | DE LUC'S HYG. | | WIND. | | ATMOSPHERIC VARIATIONS. | | |
|---|---|---|---|---|---|---|---|---|---|---|---|---|---|---|
| | | | 9 A.M. | MAX. | MIN. | 9 A.M. | 10P.M. | 9 A.M. | 10P.M. | 9 A.M. | 10P.M. | 9 A.M. | 2 P.M. | 10P.M. |
| Dec | | | | | | | | | | | | | | |
| 20 | | | 48 | 48 | 46 | 29.32 | 29.28 | 84 | 84 | ESE | SE | Fine | Fine | Fine |
| 21 | | .10 | 48 | 50 | 46 | 29.34 | 29.38 | 83 | 92 | SSE | SSE | | | Sleet |
| 22 | | | 46 | 47 | 40 | 29.42 | 29.60 | 90 | 84 | WNW | WNW | Fair | | Fine |
| 23 | | .10 | 40 | 48 | 36 | 29.73 | 29.61 | 89 | 87 | W | WSW | Fine | | Rain |
| 24 | | | 37 | 41 | 41 | 29.90 | 30.11 | 84 | 84 | W | WSW | Foggy | | Fine |
| 25 | ○ | | 47 | 48 | 37 | 29.61 | 29.77 | 94 | 71 | SW | NW | Fair | | |
| 26 | | | 38 | 43 | 29 | 29.77 | 29.65 | 80 | 73 | W | N | Fine | | |
| 27 | | | 30 | 43 | 30 | 29.72 | 29.67 | 78 | 73 | WNW | WNW | | | |
| 28 | | Rain-gauge frozen. | 32 | 36 | 33 | 29.53 | 29.48 | 76 | 79 | W | W | | | Foggy |
| 29 | | | 33 | 36 | 32 | 29.46 | 29.46 | 84 | 84 | N | N | | | Fine |
| 30 | | | 33 | 34 | 28 | 29.49 | 29.55 | 90 | 87 | N | W | | | Cloud. |
| 31 | | | 29 | 34 | 28 | 29.61 | 29.65 | 87 | 84 | WSW | WSW | | | Fine |
| Jan. | | | | | | | | | | | | | | |
| 1 | ☾ | | 36 | 41 | 39 | 29.63 | 29.61 | 92 | 89 | SSE | SSE | Rain | Rain | Cloud. |
| 2 | | | 38 | 41 | 32 | 29.67 | 29.69 | 81 | 81 | SE | ESE | Fine | | Fine |
| 3 | | | 35 | 35 | 34 | 29.71 | 29.75 | 80 | 84 | E | E | | | |
| 4 | | | 35 | 36 | 35 | 29.76 | 29.76 | 81 | 81 | ENEv | ENE | | | |
| 5 | | | 36 | 36 | 35 | 29.75 | 29.71 | 82 | 92 | ENE | E | | | Rain |
| 6 | | | 36 | 36 | 35 | 29.57 | 29.61 | 92 | 92 | E | E | Rain | Rain | |
| 7 | | | 35 | 36 | 32 | 29.68 | 29.75 | 84 | 79 | ENE | ENE | Cloud. | Fine | Cloud. |
| 8 | ● | | 43 | 33 | 25 | 29.80 | 29.87 | 72 | 67 | E | NE | Fine | | Fine |
| 9 | | | 28 | 33 | 24 | 29 90 | 29.85 | 67 | 71 | ENEv | NE | | | |
| 10 | | | 25 | 30 | 29 | 29.67 | 29.55 | 76 | 78 | N | WNW | | | |
| 11 | | | 30 | 33 | 22 | 29.56 | 29.62 | 85 | 80 | NW | NW | Snowy | | |
| 12 | | | 25 | 31 | 21 | 29.65 | 29.71 | 82 | 75 | W | WSW | Fine | | |
| 13 | | | 22 | 29 | 20 | 29.81 | 29.81 | 80 | 78 | W | W | | | |
| 14 | | | 21 | 27 | 19 | 29.93 | 29.96 | 82 | 85 | W | NNE | Foggy | | Foggy |
| 15 | | | 20 | 29 | 19 | 30.12 | 30.26 | 90 | 85 | NNE | NE | | | |
| 16 | ☽ | | 20 | 28 | 20 | 30.37 | 30.41 | 85 | 85 | NE | SSE | | | |
| 17 | | | 25 | 32 | 27 | 30.44 | 30.44 | 88 | 82 | SSE | S | Fine | | Fine |
| 18 | | | 30 | 36 | 37 | 30.34 | 30.15 | 87 | 85 | SW | SW | | | |
| 19 | | | 40 | 42 | 36 | 29.97 | 29.97 | 93 | 85 | WNW | N | | | |

The Rain-gauge having frozen, no account could be taken of the quantity of Rain fallen.

